# Lipomatous hypertrophy of cardiac interatrial septum: A case report

**DOI:** 10.1097/MD.0000000000044991

**Published:** 2025-10-03

**Authors:** Sigitas Laima, Migle Pauliukonyte, Sigitas Chmieliauskas, Andrius Berukstis, Diana Vasiljevaite, Dalius Banionis, Paulius Petreikis, Edvardas Zurauskas, Donatas Petroska, Jurgita Stasiuniene

**Affiliations:** a Department of Pathology, Forensic Medicine, Institute of Biomedical Sciences of the Faculty of Medicine of Vilnius University, Vilnius, Lithuania; b Clinic of Heart and Vessel Diseases, Institute of Clinical Medicine of the Faculty of Medicine, of Vilnius University, Vilnius, Lithuania; c Department of Anatomy, Histology and Anthropology, Institute of Biomedical Sciences, Faculty of Medicine, of Vilnius University, Vilnius, Lithuania.

**Keywords:** autopsy, forensic pathology, lipomatous hypertrophy, sudden cardiac death

## Abstract

**Rationale:**

Lipomatous hypertrophy (LH) of the cardiac interatrial septum is a rare benign lesion of the heart, usually described as a nonencapsulated mass arising from the atrial septum, with common symptoms, such as atrial fibrillation, supraventricular tachycardia, and syncope. It is very important to detect LH as early as possible; however, this can be difficult due to the rarity of the condition.

**Patient concerns:**

Here, we report the unique case of a 60-year-old-man who was found to be unconscious in his car by a paramedic. The patient was declared dead after a preliminary diagnosis of unspecified sudden cardiac arrest. The patient had a history of heart disease and was referred several times by a cardiologist.

**Diagnoses:**

The final diagnosis of LH was made after autopsy.

**Interventions:**

Autopsy revealed LH of the cardiac interatrial septum, a soft, smooth mass 12 cm in length, up to 8 cm in width, and weighing 90 g. The interior of the mass was firm, nonelastic, and yellowish in color, with a few vessels. Histological examination confirmed adipose tissue proliferation and perivascular connective tissues between the cardiomyocytes and hypertrophic cardiomyocytes.

**Outcomes:**

Despite comprehensive examinations, LH was not diagnosed prior to the patient’s death, which resulted in a sudden and unexpected fatal outcome. Resuscitation efforts were unsuccessful, and the cause of death was determined postmortem through autopsy.

**Lessons:**

This case highlights the importance of considering LH as a potential etiology of sudden cardiac death, particularly in patients with known cardiac risk factors.

## 1. Introduction

Lipomatous hypertrophy (LH) of the interatrial septum is rare, accounting for only 0.6% of all cardiac tumors. It is a benign lesion of the heart that is usually found in older overweight patients. This condition usually remains asymptomatic, but can cause symptoms such as congestive heart failure, atrial fibrillation, and syncope.^[1]^ Without early diagnosis, it may lead to life-threatening conditions, such as superior vena cava syndrome, severe cardiac arrhythmias, pericardial effusion, and sudden cardiac death. Echocardiography is the primary diagnostic tool for identifying cardiac growth; however, further imaging modalities such as computed tomography (CT) or magnetic resonance imaging (MRI) may be necessary to confirm this diagnosis. Histologically, LH is a nonencapsulated mass that consist of proliferating adipose cells and hypertrophic cardiomyocytes.^[1–3]^

### 1.1. Materials and methods

This case report was approved by the Ethics Committee for Scientific Research of the Faculty of Medicine at Vilnius University. This case report was written in accordance with CARE guidelines. Written informed consent was secured from the patient’s representatives; all procedures were conducted in accordance with institutional and ethical guidelines governing postmortem examinations. An analysis was performed to evaluate data from the State Forensic Medicine Service, focusing on full forensic pathology autopsies between 2015 and 2024. During this period, there was only 1 case of LH in Lithuania, diagnosed after postmortem examination. This study has several limitations. Postmortem CT scans were not performed, and as a single case report, it describes a unique clinical situation, thereby limiting the generalizability of the findings to a broader patient population.

### 1.2. Toxicological analysis

After forensic dissection, the blood and urine samples were collected for alcohol and drug testing. Headspace gas chromatography was used to detect alcohol. Liquid chromatography-time-of-flight mass spectrometry (LC/MS-TOF) and liquid chromatography-tandem mass spectrometry (LC-MS/MS) was used for quantitative drug detection.

### 1.3. Histological methods

Histological sections were prepared for routine light microscopy analysis. Histomorphological features of the samples were examined using hematoxylin and eosin (H&E) staining. Next, Perl Prussian blue reaction was used to detect ferric iron and Masson trichrome staining of the collagen fibers. Hematoxylin and eosin staining consisted of several stages, including paraffin removal, staining, and dehydration. Sections were then deparaffinized by keeping them sequentially in absolute alcohol, 96% and 70% ethanol, and distilled water for a certain time. The specimens were stained with hematoxylin and continuously irrigated with flowing water. Subsequently, an Eosin-floxed solution was used. Finally, specimens were quickly sequentially dehydrated in 70%, 90%, and absolute alcohol, and enclosed with covering material. The nucleus and other DNA/RNA-containing structures were stained blue-violet, while the cytoplasm and matrix were stained pink.

## 2. Case report

**Figure 1.**
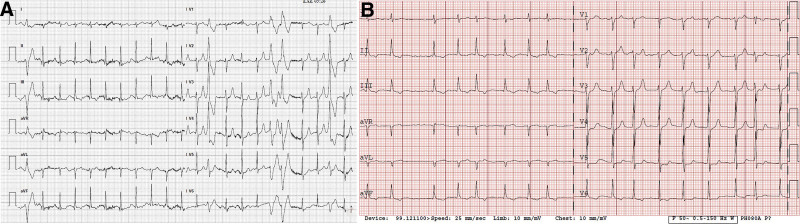
Electrocardiogram.

**Figure 2.**
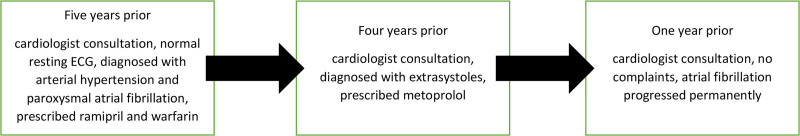
Summary of premortem clinical data.

A 60-year-old-man was found to be unconscious in his car by a paramedic. Emergency cardiopulmonary resuscitation was performed, and the patient was declared dead, with a preliminary diagnosis of unspecified sudden cardiac arrest. The patient had a cardiac history with several consultations with a cardiologist. However, 5 years prior, the patient had consulted a cardiologist; he had no complaints and had a normal resting ECG. The patient was diagnosed with arterial hypertension and paroxysmal atrial fibrillation and treated with ramipril and warfarin. During the visit, the patient underwent a stress test and did not experience chest pain, without any changes in the ST segment. However, there were ventricular extrasystoles, but few ventricular couplets (Fig. 1A). Hence, the next follow-up was scheduled after 1 year, and metoprolol was prescribed for the extrasystoles. The last time the patient visited was 1 year before his death and did not verbalize any complaints, but his atrial fibrillation progressed permanently (Fig. 1B). Echocardiography revealed a dilated left ventricle measuring 6.6 cm, left ventricular hypertrophy (intraventricular septum, 1.4 cm, posterior wall, 1.3 cm), dilated atria, and mitral and tricuspid regurgitation. These changes were interpreted as a consequence of hypertension and atrial fibrillation and did not warrant further investigation (Fig. 2).

The body was taken to the State Forensic Medicine Service of Vilnius Department of Medicine. Autopsy was performed to determine the cause of death (Table 1). First, external examination was performed. No atypical findings were observed during external examination, and there was no evidence of mechanical injury. Subsequently, an internal examination, including that of the abdominal cavity, was performed, and venous stasis was observed in the organs, indicating acute cardiopulmonary failure.

**Table 1:** Preanalytical variables by BRISQ guidelines.

I. Pre-acquisition	
Biospecimen type	Solid tissue obtained from a human being
Anatomical site	Heart samples
Biospecimen disease status	Solid tissue obtained from the disease site and from normal adjacent tissue (not involved but collected from the same anatomical site as the disease specimen in the same patient)
Clinical characteristics of patient	Atrial fibrillation and hypertension
Vital state of patient	Postmortem biospecimens were obtained
Disease state	1 year before death atrial fibrillation progressed permanently; hypertension; echocardiography revealed a dilated left ventricle, left ventricular hypertrophy, dilated atria, and mitral and tricuspid regurgitation
Cause of death	The preliminary diagnosis – unspecified sudden cardiac arrest
Agonal state	The patient was found unconscious by a paramedic; emergency CPR was performed, but the patient was subsequently declared dead.
Diagnosis	
Clinical	The preliminary diagnosis – unspecified sudden cardiac arrest
Pathologic	Lipomatous hypertrophy of the interatrial septum
Time between diagnosis and sampling	An autopsy was performed 1 d postmortem to determine the cause of death
Patient demographic information	60-year-old-man
II. Acquisition	
Collection mechanism	Autopsy was performed; solid tissue obtained from a human being
Time form cessation of blood flow in vivo to biospecimen excision/ acquisition	An autopsy was performed 1 d postmortem (28 h from cessation of blood flow in vivo to biospecimen excision/ acquisition)
Time from biospecimen excision/acquisition to stabilization	Solid tissues obtained during autopsy were promptly stabilized with formalin (28 h from death to biospecimen stabilization)
Temperature between biospecimen excision/acquisition and stabilization	20 to 25 °C
III. Stabilization/preservation	
Mechanism of stabilization	Formalin fixation
Type of long-term preservation	Formalin fixation
Constitution of preservative	10% neutral-buffered formalin
Specimen size	Cubes approximately 1 cm on a side
IV. Storage/transport	
Storage temperature	20 to 25 °C
The time or range thereof between biospecimen acquisition and distribution or analysis	1 to 2 wks
Shipping temperature	20 to 25°C
Shipping duration	1 to 2 d
Type of transport container	premanufactured shipping container
V. Quality assurance measures relevant to processing prior to analyte extraction and evaluation of the extracted analyte	
Composition assessment and selection	
Gross and microscopic review	The anatomical characteristics of the biospecimens in the study were evaluated by a forensic pathologist and a pathologist
Proximity to primary pathology of interest	Solid tissue obtained from the disease site and from normal adjacent tissue (not involved but collected from the same anatomical site as the disease specimen in the same patient)
Method of enrichment for relevant components	Histomorphological features of the samples were examined using hematoxylin and eosin (H&E) staining
Embedding reagent/medium	Paraffin

CPR = cardiopulmonary resuscitation.

**Figure 3.**
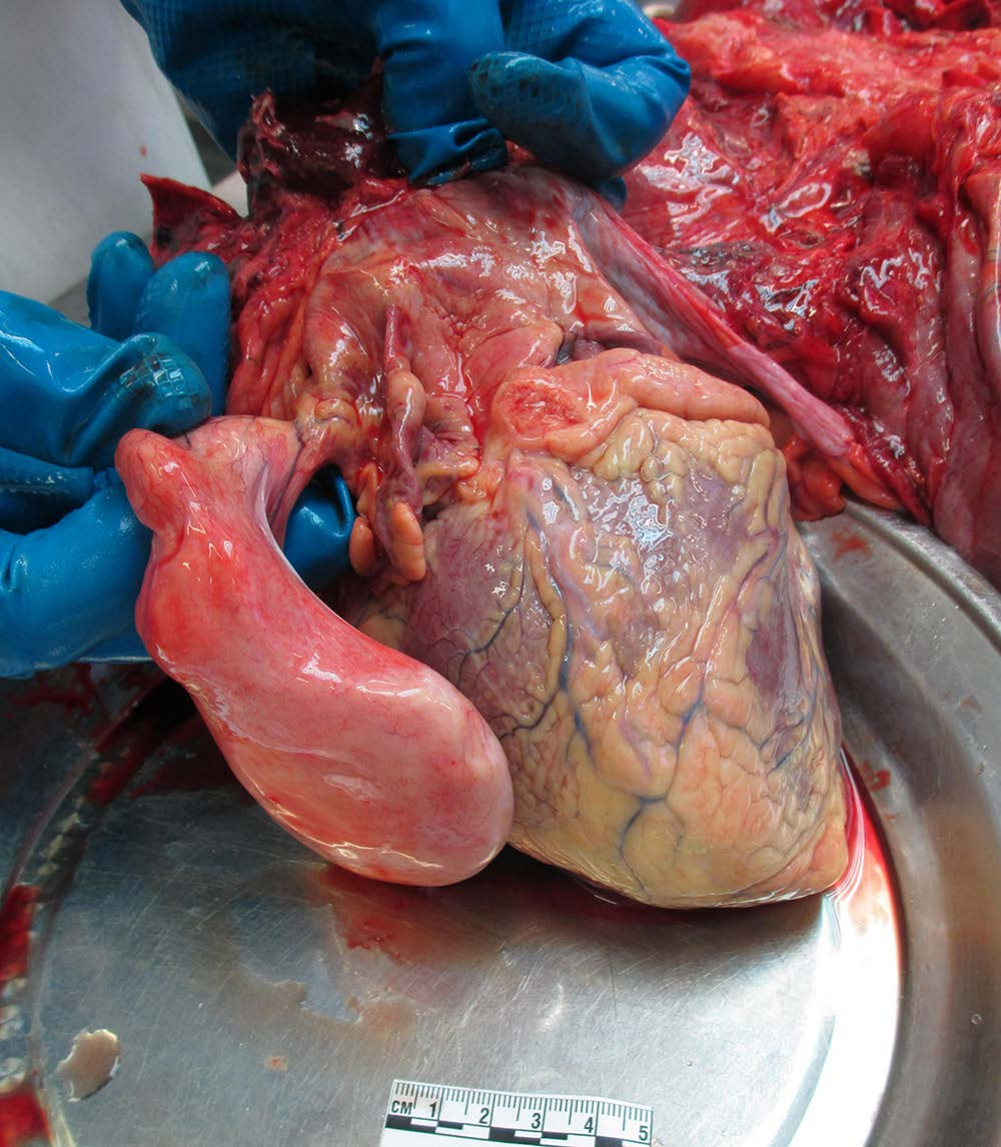
LH of interatrial septum. LH = lipomatous hypertrophy.

**Figure 4.**
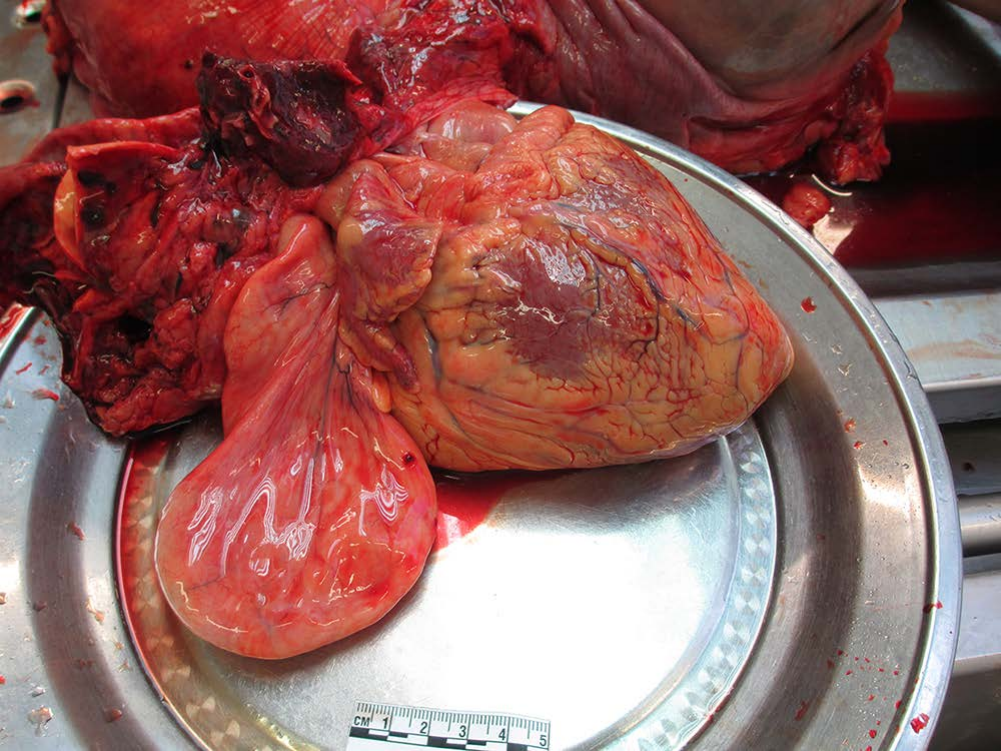
Lipomatous hypertrophy of interatrial septum and its stalk.

**Figure 5.**
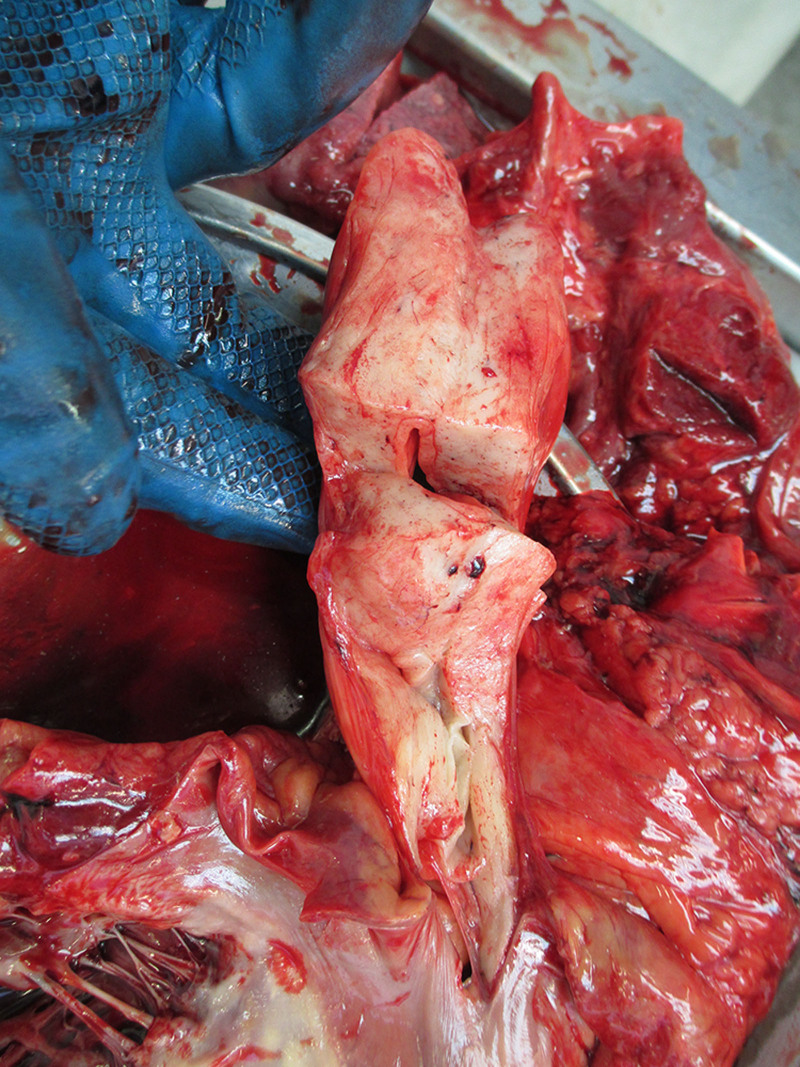
Stalk of the growth.

**Figure 6.**
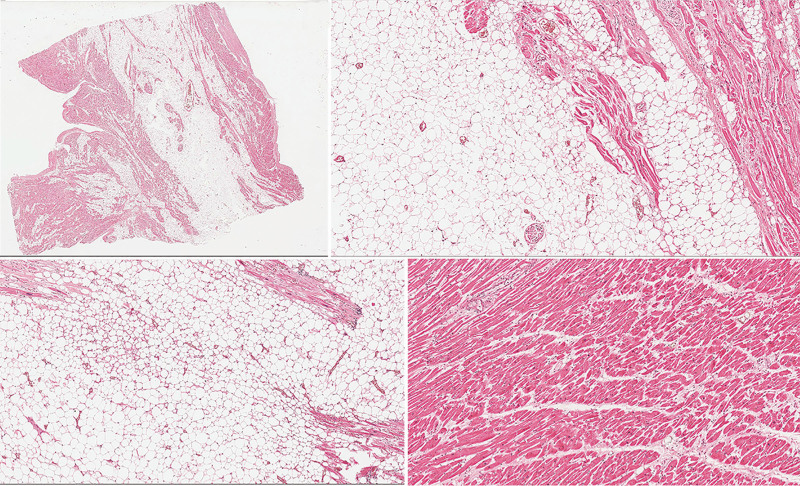
Full view of histological slide demonstrating perivascular proliferation of adipose and connective tissues among cardiomyocytes and hypertrophic cardiomyocytes. Adipose tissue was predominant in certain areas. Images were individually processed using Adobe Photoshop, including adjustments to white balance and contrast, as well as format conversion.

Next, the mediastinum was examined and 20 mL of yellow transparent fluid was found in the pericardial cavity. Atypical changes were not observed in the visceral pericardium. While inspecting the heart, the examiners found that the dimensions of the heart were 6 × 13 × 7 cm, with a weight of 570 g. The stalks were attached near the left outer part of the interatrial septum (Fig. 3). It was pear-shaped, firm, smooth, and yellowish in color (Fig. 4). It weighed 90 g, was 12 cm long, and was up to 8 cm wide. The interior of the growth was smooth, nonelastic, and contained few vessels (Fig. 5). Continuing the heart inspection, the adipose tissue around the heart was up to 1.5 cm in width, covering 75% of the heart’s surface. Calcified atherosclerotic plaques were observed in the inner part of the coronary artery wall, obstructing up to 50% of the diameter of the vessel, and atherosclerotic plaques in the aorta, obstructing ¼ of its diameter. In addition, some non-coagulated blood was found in the heart chambers, which could indicate acute cardiac failure. Other significant findings included pulmonary and brain edema. No other abnormalities were observed, indicating a cause of death. Additionally, blood and urine toxicological tests were performed; however, the results were negative. Moreover, histological examination of interatrial growth was performed (Fig. 6). There was perivascular proliferation of adipose and connective tissues and between cardiomyocytes and hypertrophic cardiomyocytes. Diffuse proliferation of adipose connective tissue was observed throughout the entire atrial septum. Adipose tissue is dominant in some areas. It is important to note that no atypical adipose cells were seen.

LH of the interatrial septum is a primary cause of death. Complications included acute cardiopulmonary failure, judging by the presence of non-coagulated blood in the heart chambers, venous stasis of the internal organs, and signs of pulmonary and brain edema. Additional findings included III-stage coronary artery atherosclerosis with 50% stenosis and stage IV aortic atherosclerosis.

## 3. Discussion

LH is a rare cardiac disorder. It was first described in 1964, and, as of 2006, approximately 200 cases have been documented.^[1]^ Mostly, this condition is asymptomatic, but it can cause symptoms such as heart palpitations, dizziness, atrial fibrillation, syncope, congestive heart failure, or even more dangerous conditions, such as superior vena cava syndrome, severe cardiac arrhythmias, pericardial effusion, and sudden cardiac death.^[2–5]^ If a patient has a history of coronary artery disease and angina, LH can present with chest pain similar to angina.^[6]^ In the present case, the patient was diagnosed with permanent atrial fibrillation and died of sudden cardiac death caused by LH induced by ventricular arrhythmia.

Histologically, this lesion is usually described as a nonencapsulated mass that mostly consists of adipose tissue and can include hypertrophied cardiomyocytes. Myocytes often vary in shape and size; however, no signs of malignancy have been observed. Collagen bands and chronic inflammatory cells were also observed.^[1,5,7,8]^ In some cases, there may be a mixture of mature and brown adipose tissue.^[1,7]^ The lesion cells may also have a microvacuolated cytoplasm and centrally located nuclei of various sizes.^[8]^

It is usually found accidentally during echocardiography, CT, or MRI. Echocardiography is one of the most popular primary diagnostic tools for identifying cardiac growths. However, using this method, it is difficult to differentiate the type of lesion and whether it is benign or malignant. A multi-slice CT scan is very useful in the differentiation of LH, as this growth has specific characteristics that are clearly seen in the images.^[9]^ More specific characteristics of LH can be observed on MRI. It can appear as a high-signal pear-shaped mass on T1-weighted images, and its intensity decreases with fat-suppression sequences, which is a characteristic of fat. LH is usually diagnosed based on CT or MRI results, and no biopsy is required.^[10]^ In the present case, transthoracic echocardiography revealed hypertrophy of the intraventricular septum and atrial enlargement. However, no further testing was performed to accurately diagnose the condition, as these changes were interpreted as a result of atrial fibrillation and arterial hypertension.

The differential diagnosis of LH most commonly includes lipomas and myxomas. Lipoma and LH appear very similar on CT and MRI scans because they mainly consist of adipose tissue; however, the main differentiating characteristics of lipomas are that they are encapsulated and do not infiltrate atrial muscle cells.^[11]^ Myxomas are similar in shape but usually consist of typical myxoma cells and myxoid areas. The most notable difference is the lack of fat, which can be observed on CT and MRI.^[5,12]^

The treatment of this lesion depends on the patient’s symptoms. If the growth is small and the patient is asymptomatic, treatment is not necessary.^[13]^ The most common symptom of LH is atrial arrhythmia, which is typically treated with antiarrhythmic agents. In some cases, this lesion may present with more dangerous arrhythmias, such as sick sinus syndrome. Therefore, a cardiac pacemaker should be implanted.^[14]^ Rarely, LH can cause dangerous arrhythmias or disrupt blood flow to and from the heart by mechanically obstructing circulation. In 1 patient, LH caused right atrial inflow obstruction by suppressing the superior vena cava. Surgical resection is the best approach for the treatment of mechanical obstructions. In this case, the obstruction was elevated by the resection of the hypertrophy, which resulted in better control of the patient’s atrial flutter.^[15]^

The mechanism underlying arrhythmias associated with LH remains unclear. This may be because of the infiltration of adipocytes into the myocardium near the atrioventricular nodes. Infiltration can obstruct the electrical conduction system and disrupt the flow of electrical signals, which can cause arrhythmias.^[16]^ Dangerous cardiac arrhythmias may arise because of extensive bleeding from the tumor.^[4,17]^ Arrhythmias can also be caused by the mechanical pressure on the electrical conduction system. It most commonly occurs during cardiac procedures, such as catheterization, and if LH causes mechanical pressure similar to that of the conduction system, it could cause arrhythmias.^[18]^ As LH is associated with older, obese patients, coronary artery disease could provoke arrhythmias by causing ischemic damage to the atrioventricular node and electrical conduction system.^[4]^

Most recently published research shows that LH of the interatrial septum is usually an incidental finding and is asymptomatic, although it may be associated with atrial arrhythmias. The primary differential diagnosis is cardiac lipoma, which is distinguished by its encapsulation and sparse myocyte content. Cardiac MRI remains the gold-standard diagnostic tool. Most patients with findings typical of LH of the interatrial septum on advanced imaging, without evidence of structural or arrhythmic complications, can be managed conservatively. Surgical management is generally limited to cases in which LH of the interatrial septum is complicated by serious arrhythmias, superior vena cava syndrome, right atrial obstruction, or hemodynamic derangements, leading to congestive heart failure.^[19,20]^

Cardiac MRI is the most effective imaging modality for delineating tumor borders and assessing their extension into the interventricular septum and ventricular free wall. Patients require close monitoring because LH of the interatrial septum may lead to right or left ventricular outflow tract obstruction or superior vena cava obstruction, potentially necessitating cardiac surgical intervention.^[21–24]^

The term LH of the interatrial septum is slightly misleading, as histologically, the lesion results from the proliferation of fat cells rather than hypertrophy. The use of advanced diagnostic tools significantly improves the diagnostic accuracy, enabling better differentiation between LH and other cardiac masses or pseudomasses.^[25]^

LH of the interatrial septum is characterized by excessive adipose tissue deposition (>2 cm) in the atrial septum, typically sparing the fossa ovalis and presenting as a characteristic hourglass-shape. Unlike cardiac lipomas, adipose tissue in LH of the interatrial septum is not encapsulated. The reported prevalence ranges from 2.2% in patients undergoing multi-slice CT to approximately 8% in those evaluated using transesophageal echocardiography. A higher prevalence of LH of the interatrial septum is associated with advanced age, obesity, female sex, and long-term corticosteroid use. Although LH of the interatrial septum is considered a benign finding that does not typically require specific treatment, it may lead to cardiac arrhythmias, superior vena cava obstruction, atrioventricular block, or even sudden cardiac death in some cases. The severity of these manifestations appears to correlate with lesion size and extent.^[26]^

Although LH of the atrial septum is histopathologically benign, it may also be associated with clinically significant symptoms. The mass effect can impinge on the orifices of the venae cavae, leading to variable obstruction of the systemic venous return to the heart. This may result in dyspnea, peripheral edema, and other signs of heart failure. In addition, LH of the atrial septum is associated with atrial arrhythmia.^[27]^

Excessive growth of a lipoma with myocardial infiltration may indicate a more serious clinical course and unfavorable prognosis. Subendocardial benign tumors may obstruct blood flow. In some cases, the septal wall may bulge into the left atrium, leading to a reduction in left atrial volume and subsequent pulmonary congestion. This may explain symptoms such as dyspnea, fatigue, and syncope.^[28]^

The prevalence of LH of the atrial septum in patients with chronic obstructive pulmonary disease is high (72.6%), and the adipose tissue demonstrates higher density, suggesting a greater brown fat component. In obese patients, LH of the atrial septum density is lower and decreases with increasing body mass index. Although the severity of coronary stenosis and coronary calcium levels did not differ significantly, high-risk plaques were more frequently observed in patients with severe chronic obstructive pulmonary disease.^[29,30]^

## 4. Conclusions

LH of the interatrial septum is a rare disorder that is associated with severe cardiac arrhythmia and sudden cardiac death. In this particular case, the condition resulted in atrial fibrillation and delayed diagnosis contributed to the fatal outcome of sudden cardiac death. LH of the cardiac interatrial septum can be easily diagnosed by applying and combining several imaging methods, with echocardiography typically serving as the initial diagnostic tool, followed by CT or MRI. Similarly, it is important to underscore the critical importance of early detection in clinical practice.

## Author contributions

**Data curation:** Paulius Petreikis, Edvardas Zurauskas, Donatas Petroska, Jurgita Stasiuniene.

**Writing – original draft:** Sigitas Laima, Migle Pauliukonyte, Sigitas Chmieliauskas.

**Writing – review & editing:** Andrius Berukstis, Diana Vasiljevaite, Dalius Banionis.
